# Exploring microorganism–host interactions: Emerging organoid models and analytical approaches

**DOI:** 10.1002/mlf2.70053

**Published:** 2026-02-24

**Authors:** Yue Shi, Min Xu, Yanhong Huang, Jing Qu, Shumin Liao, Yingzi Liu, Liang Li

**Affiliations:** ^1^ Joint Laboratory of Guangdong‐Hong Kong Universities for Vascular Homeostasis and Diseases, Department of Pharmacology School of Medicine, Southern University of Science and Technology Shenzhen China; ^2^ Department of Pathogen Biology Shenzhen Center for Disease Control and Prevention Shenzhen China; ^3^ Intervention and Cell Therapy Center, Peking University Shenzhen Hospital Shenzhen China

**Keywords:** disease modeling, host–microbe interaction, infectious diseases, organoids, organ‐on‐a‐chip

## Abstract

Microorganisms play a vital role in human health through their interactions with the body. Studies of host–microbe mechanisms and interactions are crucial for advancing health management. Recently, the organoid‐based models have provided new platforms in this field. Derived from human tissues, these models offer several advantages over traditional systems and, when combined with advanced analytical techniques, they enable deeper insights into host–microbe interactions. In this review, we summarize the different models and techniques used, with a particular focus on the newly developed organoid models. We discuss how these models can be effectively utilized in microorganism–host interaction studies and address their associated limitations.

## INTRODUCTION

Microorganisms, either pathogens or beneficial entities, are well recognized for their significant impact on human health. The human body hosts a diverse array of microorganisms (including bacteria, viruses, fungi, archaea, and phages), collectively referred to as the microbiome^1^. The microbiome encompasses both the microorganisms themselves and their collective genetic materials within a specific environmental niche. This microbiota is essential for a variety of physiological functions, including metabolism, barrier integrity, immune response, and systemic organ signaling^2^.

The interactions between microorganisms and host cells are crucial to human health. Recent advances in microbiome research have linked disturbances in these interactions to a broad spectrum of conditions, ranging from chronic inflammatory and metabolic disorders to neurological diseases and cancers^3^. Pathogens, microorganisms that can cause diseases, have shaped human history and remain as a persistent threat^4^. Research into host–pathogen interaction is rapidly evolving, providing insights into disease pathogenesis and informing potential therapeutic strategies to address them. The continuous emergence and resurgence of infectious diseases highlight the global challenges posed by virulent pathogens. Deciphering host–pathogen interactions is crucial for understanding both host defense mechanisms and pathogen strategies, providing the foundation for developing innovative approaches to combat and control the spread of infectious diseases.

A critical question in this field is how both pathogenic and non‐pathogenic microorganisms interact with the human host. Pathogens must adapt to their environment, altering gene expression and metabolic pathways essential for their survival^5^. Environmental factors, such as culture conditions, host physiology, and immune responses, also significantly influence microbial behavior. Therefore, accurate studies of host–pathogen interactions necessitate a careful selection of experimental models.

This review will outline the models and techniques used in microorganism–host interaction studies, with a specific emphasis on the utility and current knowledge of organoid models. We will discuss how organoids have advanced research in this domain and address the limitations that currently constrain the broader application of this approach. Viruses, due to their distinctly different nature from bacteria and fungi and the length limitation of this review, will not be covered in this review.

## TRADITIONAL MODELS USED IN MICROORGANISM–HOST INTERACTION STUDIES

### Cell line cultures

Cell line cultures are established models for studying infections due to their standardized setups, reproducibility, ease of handling, and cost‐effectiveness. They are therefore also suitable for high‐throughput screening. Various human body organs can be modeled using different cell line types, as detailed in Table 1. However, 2D cell cultures present limitations, such as a limited variety of cell types, altered physiology compared to tissue cells, and a lack of intercellular communication, which are crucial for accurate organ representation^100^, ^101^.

**Table 1:** Summary of common microbes related to different organs, and the use of cell lines and *in vivo* animal models in studying host–pathogen interactions.

	Microbes	Cells	Animal models	References
**Skin**	Group A *Streptococcus* (*Streptococcus pyogenes*), *Trichophyton rubrum*, *Staphylococcus aureus*, *Klebsiella pneumoniae* , *Enterobacter aerogenes* , *Proteus vulgaris* , *Coryneform bacteria* , *Mycobacterium tuberculosis* , *Mycobacterium marinum* , *Propionibacterium acnes* , and *Mycobacterium ulcerans*	HaCaT, HEK293T, pooled neonatal normal human epidermal keratinocytes, fibroblast GM07492, N/TERT‐2G, corneocytes, PHKs, and HFF‐1	HSD:ICR mice, CD‐1 (S1c:ICR) mice, C57BL/6 mice, guinea pigs, and BALB/c mice	6, 7, 8, 9, 10, 11, 12, 13, 14
**Brain**	*Streptococcus pneumoniae*, *Neisseria meningitidis*, *Streptococcus agalactiae*, *Escherichia coli*, *Listeria monocytogenes*, *Haemophilus influenzae* , and *Klebsiella* spp.	iBECs, meningioma cells, trigeminal neurons, and HBMECs	C57BL/6 mice, Wistar rats, New Zealand white rabbits, Nav 1.8‐Cre, mice, *Ramp1* ^*fl/fl*^ mice, *Calca* (CGRPα)‐GFP‐DTR^flox^ mice, and *Galleria mellonella*	15, 16, 17, 18, 19, 20, 21, 22
**Respiratory tract**	*Pseudomonas aeruginosa, Streptococcus pneumoniae, Haemophilus influenzae, Mycobacterium tuberculosis, Staphylococcus aureus, Streptococcus pyogenes, Moraxella catarrhalis, Neisseria subflava*, and *Pneumocystis carinii*	Calu‐3, HBE cells, BEAS‐2B, CF15, A549, Hep‐2, HAECs, NHBE cells, H292, AECII, Calu‐6, SAECs, NCI‐H441, TT1, and hAELVi	F344 rats, BALB/c mice, C3H mice, C57BL/6 mice, rhesus macaques, cotton rats, Sprague‐Dawley rats, OT‐II TCR transgenic mice, CD‐1 mice, baboon, and CBA mice	23, 24, 25, 26, 27, 28, 29, 30, 31, 32, 33, 34, 35, 36, 37, 38, 39, 40, 41, 42, 43, 44, 45, 46, 47, 48
**Gastrointestinal tract**	*Yersinia enterocolitica, Acinetobacter baumannii, Escherichia coli, Salmonella* spp., *Shigella flexneri, Clostridioides difficile, Fusobacterium nucleatum, Campylobacter jejuni, Vibrio cholerae*, and *Helicobacter pylori*	Caco‐2, HT‐29, HCA‐7, HEK293T, HeLa, U937, T84, Henle‐407, HCT‐8, MEFs, HCT116, AGS, CHO K1, GD 25, HL‐60, GE 11, and MKN‐28	C57BL/6 mice, Holstein calves, CD‐1 mice, 129/SvEv mice, gnotobiotic piglets, C3H mice, guinea pigs, rhesus macaques, and *Mongolian gerbils*	49, 50, 51, 52, 53, 54, 55, 56, 57, 58, 59, 60, 61, 62, 63, 64, 65, 66, 67, 68, 69, 70
**Urinary tract**	*Klebsiella pneumoniae, E. coli, Pseudomonas aeruginosa, Acinetobacter baumannii, Staphylococcus saprophyticus, and Enterococcus faecalis*	5637, HUCs, and MB49	C3H mice, *Galleria mellonella*, C57BL/6 mice, CBA mice, BALB/c mice, CF‐1 mice, and Parkes mice	71, 72, 73, 74, 75, 76, 77, 78, 79, 80
**Reproductive system**	*Chlamydia trachomatis*, *Lactobacilli*, *Neisseria gonorrhoeae*, *Staphylococcus* spp., *Streptococcus* spp., *E. coli* , *Enterococcus faecalis* , *Mycoplasma* spp., *Klebsiella pneumoniae* , and *Candida *spp*.*	HeLa‐229, A2EN, VK2, HeLa, McCoy, BMDCs, THP1, hEECs, HEC‐1‐B, T84, and PBMCs	BALB/c mice, C57BL/6 mice, and NSG mice	81, 82, 83, 84, 85, 86, 87, 88, 89, 90, 91, 92
**Heart**	*Staphylococcus*, *Streptococcus*, *Enterococcus*, *Bacilli*, and *Candida*	Infective endocarditis vegetation, HUVECs, and EA.hy926	C57BL/6 mice, Wistar rats, piglets, and Sprague‐Dawley rats	93, 94, 95, 96, 97, 98, 99

Microbes with underlined species names have been documented in clinical findings, but few experimental studies have been conducted to investigate the underlying mechanisms in specific systems. AECII, alveolar epithelial cells type II; BMDC, bone marrow‐derived dendritic cell; HAEC, primary human airway epithelial cell; HBE, human bronchial epithelial cells; HBMEC, brain microvascular endothelial cell; hEEC, primary human endometrial epithelial cell; HUC, human uroepithelial cells; HUVEC, human umbilical vein endothelial cells; iBEC, iPSC‐derived brain microvascular endothelial‐like cell; MEF, mouse embryonic fibroblast; NHBE, primary human bronchial epithelial cells; PBMC, peripheral blood mononuclear cell; PHK, primary human keratinocyte; SAEC, small airway epithelial cells.

### *In vivo* models

Traditionally, *in vivo* models have been considered the benchmark for studying infectious diseases, enabling observation of organ‐specific effects and systemic immune responses. Various animal models have been developed based on infection sites (Table 1). Yet, discrepancies in species biology often challenge the translation of results into human conditions. Humanized mouse models offer a partial solution but are limited by immune deficiencies, high cost, and high individual variability, complicating their use, especially in high‐throughput or large‐scale studies.

### Precision‐cut tissue slices or tissue samples

Precision‐cut tissue slices serve as a bridge between traditional cell cultures and *in vivo* animal models because they can accurately reflect the cellular and structural complexity of the tissue *in situ*. This technique allows researchers to study the microenvironment of specific organs with greater accuracy, making it valuable across various research fields, including infectious diseases, cancers, environmental toxin exposures, and organ‐specific conditions like fibrosis and genetic disorders^102^, ^103^.

However, precision‐cut tissue slices have notable limitations. First, their availability is often constrained due to ethical concerns. Additionally, they lack immune components, which can significantly influence infection dynamics. Another challenge is that the structural integrity of the slices can deteriorate over time, potentially affecting their functional stability and altering infection patterns. Their primary limitation, though, is their short functional lifespan: intestinal slices remain viable for only a few hours^104^, while liver slices can be maintained for up to 2 weeks under optimized conditions, though they may still undergo functional changes during culture^105^. Everted sacs, commonly used to study drug metabolism and absorption, also suffer from limited viability, typically lasting just a few hours^106^.

## ORGANOIDS AS A NOVEL PLATFORM FOR STUDYING MICROORGANISM–HOST INTERACTION

**Figure 1:**
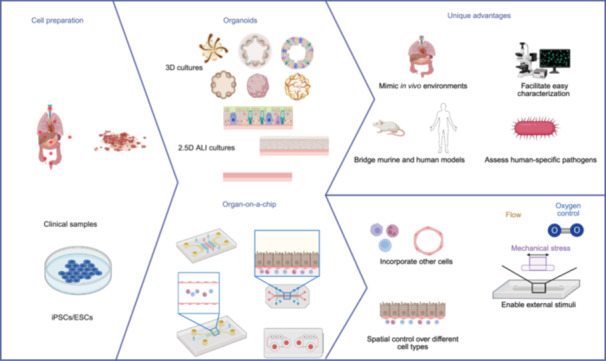
Schematic illustration of organoids and organ‐on‐a‐chip (OOC) system construction and their advantages in studying host–microbe interactions. ALI, air–liquid interface; ESC, embryonic stem cell; iPSC, induced pluripotent stem cells. This figure was created in Bio‐Render. Li, L. (2026) https://BioRender.com/jb8i58q.

Organoids are three‐dimensional *in vitro* cultures derived from stem cells or organ‐specific progenitors, which undergo self‐organization to mimic the architecture and functionality of *in vivo* organs^107^, ^108^. They can originate from somatic stem cells, induced pluripotent stem cells (iPSCs), or clusters of tissue‐specific progenitor cells. The techniques used to develop organoids are tailored to each organ type and the specific source of cells^107^, ^108^, allowing for the creation of increasingly complex and life‐like organ structures^109^, ^110^. Various organoids representing key human and mouse organs such as the brain, lung, skin, heart, vasculature, intestine, liver, spleen, and kidney have been established. In recent decades, the field of organoid research has expanded dramatically, leading to the development of organoids and their related application research. Organoids have been proven to be invaluable in a variety of fields, including developmental biology, disease modeling, cancer research, drug development, and tissue engineering^110^, ^111^, ^112^, ^113^, ^114^. In the field of microorganism–host interaction, while traditional models provide certain insights, organoids allow for a more nuanced exploration of these interactions under controlled, yet realistic conditions (Figure 1).

One of the primary advantages of organoids is their capacity to recapitulate *in vivo* environments. For example, skin organoids include both proliferative epidermal stem cells and differentiated cells, closely mimicking the human epidermis structure^115^. Similarly, respiratory organoids, including nasal, bronchial, and lung organoids, have shown that these models can replicate the specific cell types found in their *in vivo* counterparts^116^, ^117^, ^118^. When using air–liquid interface (ALI) culture methods, both skin and respiratory organoids can mimic the native tissue architecture^116^, ^117^, ^118^, ^119^. This capacity to better model the *in vivo* microenvironment makes them well suited for microorganism–host interaction studies. In ALI‐cultured respiratory organoids, as an example, the infections could occur from the apical side, while epithelial nutrient acquisition occurs via the basolateral compartment, mirroring the *in vivo* conditions^120^. This physiologically relevant setup allows infection studies using organoids to align more closely with clinical findings and to more faithfully reproduce the key disease hallmarks^121^, ^122^.

As an *in vitro* model, organoids offer an accessible platform for the observation and characterization of the infection process with high spatiotemporal resolution. They enable timely and detailed analysis of the pathogen invasion process, which animal models often struggle to achieve. Compared with *in vivo* systems, organoids also provide greater control over experimental design, allowing for the exclusion of inflammatory effects or other microenvironmental factors, thereby making them ideal for studying direct interaction between bacteria and host cells. For example, the effects of the probiotic *Lactobacillus reuteri* D8 on intestinal epithelial regeneration, homeostasis, and damage repair can be studied using organoids. Unlike the Caco‐2 cell line, organoids contain multiple cell types; unlike mouse models, they isolate the effects of bacteria on epithelial cells without interference from other microbes or cell functions^123^.

Organoids also serve as a bridge between murine and human studies. Translating drug efficacy data from mice to humans can be challenging, since murine infections often involve significantly higher quantities of log‐phase bacteria than would occur in human infections, complicating pharmacokinetic comparisons^124^. Organoids offer a more physiologically relevant infection condition. Besides, they enable validation of mechanistic findings from animal studies. For example, *Pseudomonas aeruginosa* (PA) infection in mice was found to trigger ExoU‐dependent alarmin and peroxidized lipid production. This mechanism was confirmed in human bronchial organoids, where cellular peroxidized phospholipids enhanced ExoU phospholipase activity, driving necrosis^125^.

Moreover, organoids are particularly valuable for studying human‐specific pathogens due to the lack of suitable animal models, and for microbes that require particular host cell types for replication. One example is *Moraxella catarrhalis*, a bacterium originally considered a commensal of the upper respiratory tract, but now recognized as a pathobiont. Animal models are not effective for studying this bacterium^126^, but human‐derived respiratory tract organoids have allowed researchers to explore its interactions with epithelial cells under various conditions, including viral co‐infections. They found that viral infection can enhance *M. catarrhalis* survival and its adhesion to epithelial cells^127^. Another example is *Akkermansia muciniphila*, which requires animal‐derived mucin as an essential nutrient. Normal cell lines, undifferentiated intestine organoids, and *ATOH1*‐knockout organoids lacking goblet cells failed to support its growth^128^.

Here, we summarize examples of how organoids have been applied to study host–microbe interactions, presenting them in order of the quantity of microbial load (Table 2).

**Table 2:** Brief summary of organoids used as models for host–microbe interaction studies, including common microbial strains and associated diseases for each organ.

	Organoids	Microbes	Associated disease	References
Gastrointestinal tract	Intestinal organoids	*Akkermansia muciniphila,* *E. coli, Klebsiella pneumoniae, Listeria monocytogenes, Salmonella enterica,* microbiota, and multi‐species probiotics	Intestinal infection, intestinal barrier dysfunction, colorectal cancer, and gut inflammation (e.g., inflammatory bowel disease)	129, 130, 131, 132, 133, 134, 135, 136, 137
	Gastric organoids	*Helicobacter pylori*	*H. pylori* infection and gastric cancer	138, 139, 140
Skin	Skin organoids	*Cutibacterium acnes, E. coli,* methicillin‐resistant *Staphylococcus aureus, Pseudomonas aeruginosa, Staphylococcus aureus, Trichophyton rubrum,* and multi‐strain biofilm	Nail and skin infection, and chronic infection	115, 119, 141, 142, 143, 144
Respiratory tract	Nasal organoids, bronchial organoids, lung organoids, airway organoids, and patient‐derived airway organoids	Non‐typeable *Haemophilus influenzae*, *Haemophilus influenzae, Mycoplasma, Mycobacterium abscessus, Mycobacterium tuberculosis,* non‐tuberculous *Mycobacteria, Moraxella catarrhalis, Pseudomonas aeruginosa, Streptococcus agalactiae,* and *Staphylococcus aureus*	Pneumonia, pulmonary tuberculosis, cystic fibrosis chronic obstructive pulmonary disease, chronic inflammatory lung diseases, and primary ciliary dyskinesia	120, 121, 122, 125, 127, 128, 145, 146, 147, 148, 149, 150, 151, 152, 153, 154, 155, 156, 157, 158, 159, 160, 161
Urinary tract	Urothelial organoids and bladder organoids	*E. coli, Enterococcus faecalis,* uropathogens and commensals	Bladder infection and urinary tract infection	162, 163, 164, 165, 166
Reproductive system	Fallopian tube organoids	*Chlamydia trachomatis*	*Chlamydia trachomatis* infection	167, 168, 169
Brain	Blood–brain barrier organoids	*Borrelia burgdorferi sensu lato* spirochetes	Lyme neuroborreliosis	170, 171

### Gut organoids

The relationship between bacteria and gut has long been recognized. The gut microbiota, predominantly commensal or mutualistic, exists in vast numbers and is influenced by factors such as diet, age, lifestyle, medication, and host genetics. These microorganisms play a crucial role in training host immunity, impacting both health and disease conditions^172^. Studying the interactions between specific pathogens or the microbiota community and the host is invaluable for understanding disease and health. However, unraveling the molecular mechanisms governing mucosal tissue microenvironmental responses remain challenging. Intestinal organoids faithfully recapitulate key cellular components of native intestinal tissue, including mature enterocytes, Paneth cells, goblet cells, enteroendocrine cells, and tuft cells^173^. This makes them a unique *in vitro* platform for studying host responses and bacterial activities. Intestinal organoids enable detailed characterization of the invasion processes, unraveling underlying mechanisms, and validating findings observed *in vivo*.

*Salmonella enterica* and *Listeria monocytogenes* are common pathogens that cause intestinal infections. They invade intestinal epithelial cells using different entry methods, targeting the apical and basolateral sides, respectively, and exit the epithelium via apically extruding enteroid cells. With intestinal organoids, this invasion process and early epithelial host responses can be readily captured and characterized^129^, ^130^. Similarly, organoids enable clear observation of *Klebsiella pneumoniae* invasion process, which induces pore formation in intestinal organoids. This correlates with *in vivo* barrier disruption, providing insights into the role of intestinal barrier dysfunction in the pathogenesis of primary sclerosing cholangitis^131^.

In exploring the role of genotoxic *Escherichia coli* in the occurrence of oncogenic mutations, a direct bacteria–organoid interaction environment was established by microinjecting pks^+^ *E. coli* (carrying a pathogenicity island pks^+^ encoding colibactin synthesizing enzyme) into colon organoids. After a 5‐month pathogen–organoid co‐culture, *E. coli* directly induced distinct mutational signature related to colorectal cancer in host epithelial cells^132^. Similarly, when studying the direct role of intestinal epithelial cells in restricting pathogens, organoids derived from healthy human donor and patients were compared. Organoids derived from patients with infantile inflammatory bowel disease lacked IL10RB and were unable to respond to cytokine IL‐22. It was observed that IL‐22 primed organoids from a healthy human donor effectively restricted *Salmonella enterica* infection, while this antimicrobial response was absent in patient‐derived organoids but could be restored by genetically complementing the IL10RB deficiency^133^.

Organoids offer an accessible platform for validating *in vivo* effects, such as confirming the repair effects of the *A. muciniphila* BAA‐835 strain on gut damage. In mice, it was evidenced by reduced weight loss and tissue damage via hematoxylin and eosin (H&E) staining. While in organoids, it can be easily characterized by changes in the size and number of gut organoids under optical microscopy^134^.

One unique aspect of the intestine is its microbiome environment, comprising a diverse microbiota community and a low oxygen level. An *in vitro* model suitable for the examination of intestine–microbiome interaction is important for both fundamental and applied studies. Direct co‐culture of microbiota with organoids can determine whether shifts in microbiota are a cause or a consequence of gut inflammation. Research has shown that it is the microbiota, rather than epithelial cells, that induced stress response^135^. Besides disease‐related studies, intestinal organoids can also model the prebiotic effects on both epithelial cells and microbiota components^136^. Multi‐species probiotic therapy has been shown to enhance intestinal barrier function, aligning closely with clinical studies^137^.

**Figure 2:**
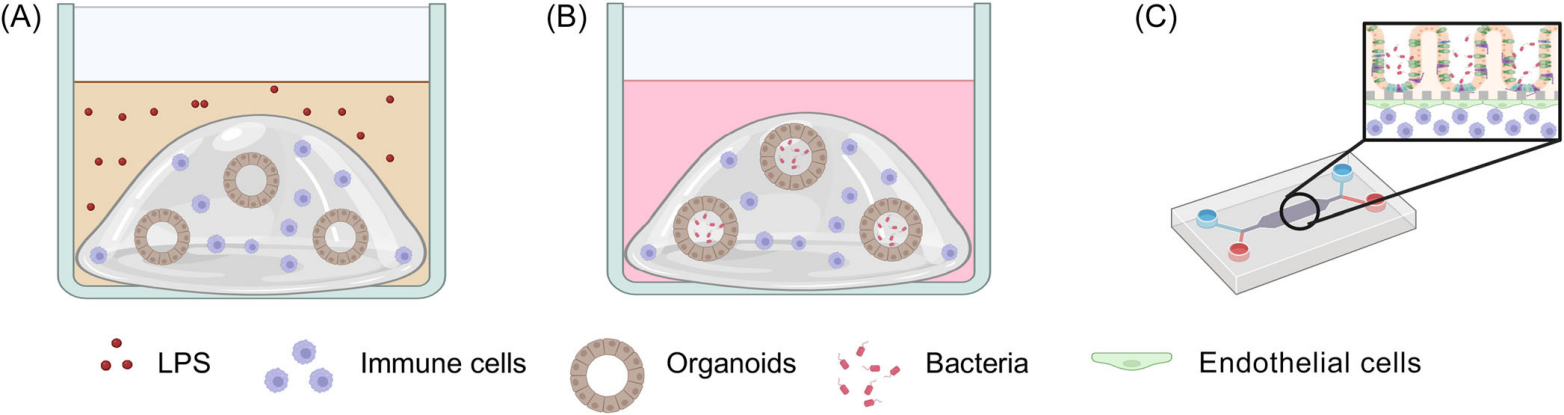
Schematic illustration of the methods used to incorporate immune cells into organoid models for bacterial infection studies. (A) Immune cells and organoids are co‐embedded in Matrigel, with inflammation modeled by adding LPS to the culture medium. (B) Bacteria are injected into the organoid lumen, and the organoids and immune cells are co‐embedded in Matrigel. (C) In an OOC model, bacteria and organoids are cultured in the upper channel, while endothelial cells and immune cells are cultured at the bottom. LPS, lipopolysaccharide. This figure was created in Bio‐Render. Li, L. (2026) https://BioRender.com/qi0i49r.

The immune response of gut epithelial organoids can be studied by co‐culturing them with immune cells (Figure 2). For example, bone marrow‐derived macrophages (BMDMs) can be embedded into Matrigel together with organoids (Figure 2A). This setup allows the modeling of inflammatory effects by using lipopolysaccharide (LPS) to study how bacterial metabolites like indoleacrylic acid (produced by *Peptostreptococcus* genus) impact the intestinal epithelial barrier under inflammatory conditions, demonstrating their ability to mitigate inflammation^174^. By combining organoids and human monocytes in Matrigel, the co‐culture system can respond to Toll‐like receptor stimulation, resulting in the release of pro‐inflammatory cytokines and the expression of tissue inflammatory markers, which in turn induces monocyte migration and differentiation, demonstrating its ability to assess the innate immune effects of various microbial ligands^175^. In addition to indirect inflammation state modeling, direct interaction between bacteria and organoids can also be achieved using a similar method (i.e., co‐embed micro‐injected organoids and macrophages into Matrigel (Figure 2B)^145^.

### Skin organoids

Skin, the body's largest organ, is vital for protection, sensation, and thermoregulation. This multilayered organ consists of the epidermis, dermis, and hypodermis, enriched with various appendages such as follicles and glands. Skin organoids have been shown to develop epidermal, dermal, and appendage components, which could replicate the complete structure and cellular composition of native skin^176^, ^177^. At the molecular level, these organoids display skin‐specific biomarkers and structures, assessed through immunofluorescent staining, and closely mirror the gene and protein signatures of the derived skin tissues^178^. Single‐cell sequencing further confirmed that skin organoids maintain major *in vivo* cellular states, showcasing their relevance to the human skin^179^. Skin organoids are commonly used to study skin infection‐related diseases such as atopic dermatitis, non‐healing wounds, and tinea pedis, providing insights into pathogen invasion dynamics, the corresponding immune responses, and drug effects.

For example, *Trichophyton rubrum*, the most prevalent dermatophyte that causes human nail and skin infections worldwide, has been shown to induce upregulation of the anti‐inflammatory factor IL‐1RN in skin organoid infection models. This response helps explain the pathogen's ability to adapt to human skin and sustain chronic infections with minimal inflammation^115^. In addition to modeling infections in healthy skin, organoids can also be used to study infections in disease models. In thermally injured skin organoids, methicillin‐resistant *Staphylococcus aureus* (MRSA) biofilms have been observed to compromise skin barrier function, promote extracellular matrix remodeling, and trigger inflammatory responses, including IL‐17, IL‐12 family, and IL‐6 family interleukin signaling, while also influencing skin metabolism^141^. These skin organoid models are useful for drug evaluation, allowing for the testing of both synthetic antibiofilm peptides like DJK‐59^141^ and commercially available drugs like vancomycin^178^.

Besides single pathogen studies, skin organoids can also be used to study the interactions among two or more pathogens. Infection with *Staphylococcus aureus* (SA) alone disrupts the skin barrier and increases the production of inflammatory cytokines, linked directly to conditions like atopic dermatitis. Pretreatment with *Cutibacterium acnes*, a commensal skin bacterium, shows a protective effect against SA infection^119^. PA is another common pathogen; the competition between PA and SA on skin organoid is influenced by host factors and shows different behaviors compared to planktonic states or biofilm assays on inert surfaces^142^. A skin organoid‐based study revealed that the two‐component regulatory system NtrBC, influenced by interspecies signaling, dictates PA's competitive edge in co‐infection scenarios^143^. In multi‐strain biofilm infection models, skin organoids enable the evaluation of the inflammatory profile of the tissue through both transcriptional and proteomic analyses of the epidermis, aiding in treatment efficacy assessments and its impact on the host. This approach is instrumental for clinicians in making informed treatment decisions by providing insights into both the biofilm's eradication and the tissue's response to treatments^144^.

One limitation of skin organoids is the absence of immune cells. While attempts have been made to integrate immune cells into skin organoids, these efforts have primarily focused on cancer modeling rather than host–pathogen interactions^176^. Additionally, the lack of a vascular system remains a significant challenge in closely replicating the *in vivo* microenvironment. However, emerging organ‐on‐a‐chip (OOC) technologies that aim to incorporate vascular systems into skin organoids have shown promise, particularly in evaluating pro‐inflammatory responses, such as those triggered b*y E. coli* infections^180^.

Beyond the absence of immune cells and vascularization, skin organoids also struggle to accurately replicate adult human skin. While skin organoids can mimic fetal skin, recreating the complexities of adult skin remains difficult. Single‐cell sequencing has revealed that skin organoids develop unique keratinocyte states, an expanded basal stem cell program, disrupted terminal differentiation, and ectopic epithelial‐to‐mesenchymal transition signatures, which diverge from their adult *in vivo* counterparts^179^. Although xenografted organoids can correct many of these *in vitro* deficiencies after transplantation, they often experience a hypoxic response that drives alternative differentiation pathways^179^.

### Respiratory tract organoids

The human respiratory tract is the primary entry point for various airborne microorganisms and particles, which are constantly inhaled and expelled^181^. Like other mucosal surfaces, the airway epithelium plays a critical role in host defense, as it directly interacts with the external environment^182^. Beyond serving as a physical barrier, airway epithelial cells function as sensors, detecting and responding to microorganisms and particles in the respiratory tract^183^. The respiratory tract is lined with two distinct types of epitheliums: airway epithelium and alveolar epithelium. The airway epithelium, which extends from the nasal cavity to the terminal bronchioles, consists of four major types of epithelial cells: ciliated, goblet, club, and basal cells. In contrast, the alveolar sacs are made up of flat type I alveolar epithelial cells and cuboidal type II alveolar epithelial cells^118^. Several respiratory diseases are associated with microbial infections, such as pneumonia, pulmonary tuberculosis, cystic fibrosis (CF), chronic obstructive pulmonary disease (COPD), chronic inflammatory lung diseases, and primary ciliary dyskinesia (PCD)‐related conditions. Respiratory tract organoids offer significant advantages for studying infection processes and evaluating therapeutic interventions, often surpassing traditional *in vitro* models^146^, ^147^.

PA and *Mycoplasma* are two common pathogens that cause pneumonia. In the study of PA infection, organoids have shown that the bacterium invades goblet cells and breaches the epithelial barrier from within. This process is promoted by cyclic di‐GMP‐dependent asymmetric division. The bacterium's Type VI secretion system facilitates preferential invasion of goblet cells, while its Type III secretion system induces goblet cell death and expulsion, leading to epithelial rupture and increased bacterial translocation to the basolateral epithelium^149^. Additionally, timely characterization of PA biofilm formation on ALI airway organoids revealed that the bacterium uses Type IV pili to contract mucus, facilitating bacterial aggregation and biofilm nucleation^150^. During *Mycoplasma* infection, it was observed that *Mycoplasma* colonization led to pericellular invasion of the basolateral compartment and migration across the transwell membrane of the ALI organoids, all while maintaining barrier function. This process, accompanied by epithelial remodeling such as cytoskeletal reorganization and deep furrow formation, provides insights into the likely routes for extrapulmonary spread observed in clinical settings, such as neural, cutaneous, and pericardial tissue fluid^120^.

In CF patients, infections caused by *Mycobacterium abscessus* (Mab) are sometimes fatal, as they usually have a greater risk of infection by the drug‐resistant non‐tuberculous *Mycobacteria* (NTM). Respiratory organoids developed from cystic fibrosis (CF) patients can support both sensitive and resistant Mabs strains while replicating CF characteristics, such as CF transmembrane conductance regulator (CFTR) channel dysfunction, mucus accumulation, and oxidative stress, making them a powerful infection model. Using this model, researchers discovered that CF conditions lead to increased Mab pathogenicity, characterized by greater bacterial growth, cording, oxidative stress, and cell death. Enhancing antioxidant pathways, particularly through the transcription factor nuclear factor erythroid‐2‐related factor 2 (NRF2)‐NAD(P)H quinone dehydrogenase 1 (NQO1) axis, may complement antibiotic treatments^121^. CF patient‐derived organoids have also been used to study the relationship between CFTR function and bitter taste receptor (T2R)‐stimulated nitric oxide (NO) responses in nasal epithelial cells, where genetically modified mouse models are often impractical. Organoids from both CF and non‐CF patients showed similar T2R expression and localization, but CF organoids showed reduced T2R/NO signaling and NO production due to CFTR dysfunction, leading to increased susceptibility to PA and other Gram‐negative bacteria that activate T2Rs^151^. Bacterial–viral co‐infections are also common in CF, particularly in patients with chronic bacterial colonization. To investigate the underlying mechanism, CF patient‐derived bronchial organoids were first infected with PA for 16 days, followed by human rhinovirus (HRV) infection. The pre‐existing PA infection altered the epithelial response to the virus, worsening inflammation and compromising barrier integrity and cell differentiation^152^.

COPD is frequently associated with infective exacerbations. Organoids derived from COPD patients show significantly enhanced pro‐inflammatory responses at both the RNA and protein levels when infected with PA and *Streptococcus pneumoniae* (SP), consistent with clinical observations^153^. Additionally, non‐typeable *Haemophilus influenzae* (NTHi) infections in COPD patients are poorly managed by macrolide antibiotics. Organoid models have revealed that COPD‐derived organoids contain abundant intracellular NTHi. Furthermore, treatment with macrolides or exposure to cigarette smoke extract impaired autophagic flux, evidenced by increased LC3‐II and sequestosome levels. These insights from organoid models are valuable for developing targeted anti‐inflammatory and antimicrobial therapies for COPD^154^.

Chronic infections, such as those caused by NTHi, are challenging to model using traditional submerged systems without antibiotics. By using ALI cultured respiratory tract organoids, it was observed that NTHi persists in the paracellular spaces, with degraded organisms found in intracellular vacuoles. While the apical surface and tight junctions remain intact, the basal cell layer shows increased junctional disorganization and wider intercellular spaces. Additionally, NTHi releases outer membrane vesicles that interact directly with host cell membranes, leading to pro‐inflammatory cytokine production, aligning with *in vivo* observations.^155^ Chronic inflammatory lung diseases could also be modeled by using TNF‐α/IL‐1β in healthy respiratory organoid cultures. The study revealed that long‐term exposure to pro‐inflammatory cytokines alters vitamin D metabolism. This alteration reduces vitamin D‐induced antibacterial activity and the production of antimicrobial peptides like hCAP18/LL‐37, due to CYP24A1 induction. This leads to reduced killing of non‐typeable NTHi, suggesting that chronic inflammation impairs vitamin D‐mediated protective responses^156^.

PCD patient‐derived organoids faithfully replicate clinical characteristics. Despite similar levels of ciliation, epithelial integrity, cytokine production, LL‐37, and nitric oxide, PCD organoids showed greater NTHi adherence and more aggregated biofilm formation compared to non‐PCD organoids^122^.

*Mycobacterium tuberculosis* (Mtb) is the leading cause of pulmonary tuberculosis. Using an organoid model, researchers examined early infection dynamics, revealing that Mtb mainly persists extracellularly and infects epithelial cells with low efficiency. In response to infection, organoids modulate the expression of cytokines, antimicrobial peptides, and mucin genes^145^. Besides, in organoids with surfactant‐deficient alveolar epithelial cells, it was found that the surfactant plays a crucial role in early Mtb infection by partially removing virulence‐associated lipids and proteins from the bacterial surfaces. At normal surfactant levels, a significant fraction of intracellular bacteria remains nongrowing, whereas surfactant deficiency leads to rapid and uncontrolled bacterial growth. This finding explains the clinical observation that smokers and elderly individuals with compromised surfactant function are at increased risk of developing active tuberculosis^157^.

In antibiotic drug efficacy testing, organoids provide a more physiologically relevant environment than 2D culture models. Some antibiotics showed enhanced efficacy in the presence of 3D lung epithelial constructs, suggesting that host factors like defensins may synergize with antibiotics to improve treatment outcomes^158^. Additionally, studies on *Haemophilus influenzae* infections revealed that biofilms formed on airway organoids show greater resistance to antibiotics compared to their planktonic states^159^.

### Urinary tract organoids

Urinary tract infections (UTIs), referring to any infection of the urethra, bladder, prostate, or kidney, are a persistent and major global health concern. Among these, bladder infection is the most prevalent. Uropathogenic *E. coli* (UPEC), *Klebsiella pneumoniae, Staphylococcus* spp., *Enterococcus* spp., group B *Streptococcus, Proteus mirabilis,* PA, and *Candida* spp. are the common pathogens that cause UTIs^184^. When investigating the prevalence and mechanisms of intracellular invasion in humans with UTIs, clinical samples have limitations. Shed infected cells do not provide insights into deeper urothelial layers, and ethical concerns make it difficult to obtain urothelial biopsies from infected patients^162^. Animal models, while useful, have drawbacks such as the structure and function of the mouse urothelium differ in several ways from those of humans^124^. Organoids derived from progenitor cells overcome these limitations by replicating key structural features and biomarkers of the human urothelium, including the presence of different cell types, correct cytokeratin‐20 expression, and urine tolerance, providing a physiologically relevant platform for studying uropathogen–host interactions across multiple species of pathogens^162^, ^163^, ^164^, ^165^.

In organoids models, infection with *Enterococcus faecalis* revealed urothelial sloughing and the formation of intracellular colonies, similar to observations in patient samples^124^. Using a 3D well‐differentiated urothelial microtissue as a host model for studies with both uropathogens and commensals, researchers observed a wide range of bacterial lifestyles and host responses. The study found that invasion, filamentation, chaining, and hijacking of exfoliating cells are common survival strategies shared by both pathogens and commensals, not necessarily correlated with virulence. For example, while the *E. coli* adhesin FimH is required for intracellular bacterial community formation, it is not essential for invasion. Urothelial cells typically expelled invasive bacteria in Rab‐LC3‐decorated structures, but highly invasive uropathogens, unlike commensals, could disrupt host barrier functions, strongly inducing exfoliation and cytokine production^162^.

The most frequent bacterial cause of UTIs is UPEC. In studying UPEC‐driven recurrent UTIs, organoids allow for dynamic monitoring of quiescent intracellular reservoirs (QIRs) and intracellular bacterial communities (IBCs). It has been observed that a solitary subpopulation of intracellular and pericellular bacteria resides in the deeper layers of the stratified bladder organoid wall, beneath the superficial umbrella cell layer. These solitary bacterial populations emerge early in the infection process, alongside the formation of IBCs in the umbrella cell layer, and are notably resistant to elimination by antibiotics and neutrophils^166^.

### Other organoids

Fallopian tube (FT) organoids have demonstrated remarkable fidelity in replicating the characteristics of *in vivo* tissue. These organoids can be stably expanded for over a year without significant changes, closely mirroring the structure and marker distribution of the *in vivo* epithelium^167^. This stability and accuracy make human FT organoids an ideal platform for studying *Chlamydia trachomatis* (Ctr) infection. A key advantage of FT organoids over traditional cell lines is their ability to support chronic Ctr infection *in vitro*. While cell lines only allow Ctr propagation for a single life cycle due to the lysis of infected cells, organoids can sustain the bacteria for extended periods. This long‐term sustainability enables researchers to observe and analyze the complex dynamic of chronic infection. During these prolonged infections, Ctr has been found to increase stemness by activating leukemia inhibitory factor (LIF) signaling, a crucial signaling of stem cell properties, and to promote age‐dependent CpG methylation^168^. Moreover, human FT organoids offer a more suitable model for studying these responses during infection, addressing the significant differences observed between human and mouse models. Research using these organoids has revealed that IFN‐γ induces the downregulation of c‐Myc, a key regulator of host cell metabolism, in a STAT1‐dependent manner. Importantly, the expression of c‐Myc can rescue Ctr from IFN‐γ‐induced persistence, providing valuable insights into the host–pathogen interaction^169^.

Shifting focus to the stomach, gastric organoids have emerged as a powerful tool for studying gastric epithelium and its interactions with pathogens. These organoids, generated from gastric stem cells and indefinitely expanded (>1 year), differentiate into specific stomach lineages. They provide a model that more closely resembles the actual gastric epithelium compared to currently used cell lines^138^. Gastric organoids have proven particularly useful for studying the interactions between host and *Helicobacter pylori* (Table 2), a well‐known gastric pathogen capable of persistently colonizing the stomach in about half of the world's population, leading to chronic infection and potentially cancer^138^. Traditional mouse models typically result only in mild gastritis that does not progress to cancer, while cell lines fail to mimic the multicellular environment of the stomach and have limited self‐renewal capabilities when using primary cells^138^. Organoids, therefore, bridge this gap by providing a more accurate and versatile model. Different gastric lineages have been observed to show distinct inflammatory responses to *H. pylori* infection^138^. Furthermore, the organoid model has aided in elucidating the molecular mechanisms underlying the relationship between infection and gastric cancer. For instance, a study involving the ATG16L1 rs2241880 allele, genotyped in subjects from various ethnic cohorts with gastric (pre)malignant lesions of varying severity, utilized *in vitro* organoid models, alongside cell line studies. This study confirmed that the ATG16L1 rs2241880 G‐allele is associated with the progression of gastric premalignant lesions and cancer^139^. Additionally, by using organoids in conjunction with an *in vivo* tumor xenograft mouse model, researchers discovered that RASAL2 plays a critical role in *H. pylori*‐induced gastric tumorigenesis^140^. This finding underscores the value of organoids in uncovering complex pathways involved in disease progression.

The blood–brain barrier (BBB) is a highly regulated interface between blood and the brain. Its primary function is to protect the brain by restricting the entry of exogenous molecules and pathogens into brain tissue. The BBB is composed of glycocalyx, endothelial cells, and a basement membrane, which contains pericytes, and astrocytic end‐feet^170^. BBB organoids can be developed by using endothelial cells, pericytes, and astrocytes, which form tight junctions and control the passage of solutes as *in vivo*. Using this model, it has been found that *Borrelia burgdorferi sensu lato* spirochetes, the causative agents of lyme neuroborreliosis, are able to invade the BBB, causing swelling of the organoids and loss of structural integrity, but without inducing significant cell death^171^.

### OOC

OOC technology has recently emerged as an advanced approach to more accurately mimic the *in vivo* environment. Using microfluidic systems, OOCs integrate living cells with controlled external stimuli to replicate the physiological conditions and functions of human organs. Models have been successfully developed for the lung, liver, kidney, intestine, skin, BBB, and other organs^185^, ^186^. This system allows the study of host–microbe interactions within a more complex environment (Figure 2).

OOC systems can incorporate immune and endothelial cells to mimic blood vessels, overcoming a key limitation of conventional organoids. For example, respiratory organoids can be further enhanced with additional cell types, such as endothelial cells, fibroblasts, and immune cells, using OOC technology. This advanced setup replicates complex infection dynamics, such as the initial host response to invasive aspergillosis. For instance, when exposed to the Δ*laeA* mutant of *Aspergillus fumigatus*, a less virulent strain, the organoid showed a stronger inflammatory cytokine response and more targeted recruitment of polymorphonuclear neutrophils (PMNs) to the infection site. Besides, this system makes it possible to study volatile interactions between pathogens, such as *A. fumigatus* and PA, in a bio‐mimetic environment, providing valuable insights into pathogen dynamics^187^. Similarly, in a bladder‐on‐a‐chip model that includes blood vessels and tissue layers, immune cells respond to UPEC infection by quickly crossing the endothelial barrier to reach the site of infection. Although these immune cells can form extracellular traps, they do not prevent the formation of IBCs. Antibiotics either delayed or failed to eliminate bacteria within IBCs, allowing the bacteria to reinfect other areas of the tissue^188^. Moreover, in a similar setting in intestine‐on‐a‐chip devices (Figure 2C), the synergistic effects of immune cells and pathogens on organoid barriers have been identified, showing that the presence of PBMCs can worsen epithelial damage caused by bacteria, even with probiotics. This was reminiscent of the clinical observations that probiotics are most beneficial for ulcerative colitis patients in the early stages^137^.

The OOC system is able to precisely control the spatial distribution of different cell types, and it can more faithfully replicate *in viv*o architecture. For example, 3D BBB organoids, although composed of the same cell types and valuable for studying the neurotropic properties of pathogens, show an “inside‐out” organization in contrast to the native structure^171^. The OOC system can overcome this limitation by specifically positioning cell types to achieve physiologically relevant alignment for infection studies^189^.

OOC systems enable precise control of external factors, such as fluid flow and hypoxia, within organoids to more accurately replicate specific *in vivo* physiological conditions. In well‐studied murine models of UTIs, UPEC undergoes a characteristic morphological transition. UPEC typically shifts from a coccoid shape to a highly filamentous form during its escape from colonized bladder epithelial cells (BECs) and then reverts to its typical rod shape to invade neighboring cells, initiating new rounds of infection. These late‐stage infection events have been observed in rodent models but not in traditional *in vitro* models such as cell line assays. In contrast, OOC systems can support the outgrowth of filamentous bacteria, their reversion to rod shapes for reinvasion of BECs, and can also simulate urine flow conditions. This model opens up new possibilities for detailed studies on the mechanisms governing late and secondary UPEC infections^190^. In gut host‐microbial studies, maintaining microbial diversity *in vitro* is challenging. It has been reported that simply microinjecting microbiota into the intestinal lumen is insufficient to sustain this diversity. A rapid decline in microbial diversity is often observed postinjection, likely due to oxygen exposure, limited nutrients, and the absence of host factors^136^. Intestine‐on‐a‐chip systems could allow control over oxygen levels, offering a long‐term co‐culture platform to sustain complex microbial communities in direct contact with living human intestinal cells, especially with those anaerobic strains^191^. This direct anaerobic co‐culture is valuable, since, for instance, the upregulation of stem cell marker genes by *Bifidobacterium adolescentis* can only be observed in a direct co‐culture system, not with conditioned medium or heat‐killed strains^128^.

Another key advantage of the OOC system is its ability to incorporate sensors for real‐time biomechanical, biophysical, and biochemical monitoring and characterization. For example, mechanical sensors can track fluid shear stress and membrane movements. Electrochemical sensors detect biochemical parameters like pH, oxygen, glucose, or even biomarkers such as cytokines, metabolites, and exosomes. Optical sensors can utilize fluorescent and luminescent markers to measure metabolite expression and cell movements^185^. Although these advanced sensors have yet to be applied in host–microbe interaction studies, they hold great potential for enabling controlled environments and real‐time monitoring for the infection process in future research.

When fabricating organoid models for host–microbe interaction studies, there are several factors beyond the choice of cell types that need to be considered depending on the experimental design, for example, the spatial organization of the organoids and the inclusion of other relevant cell types, such as immune or endothelial cells. Besides, it is also important to account for the specific microenvironment within the organoids. This includes parameters such as oxygen levels that affect bacterial growth, as well as blood, urine, or gas flow conditions. Together, these factors determine whether the model is best in a 3D culture, an ALI system, or an OOC platform.

### Limitations of organoids

Despite various efforts to improve organoid models, they still differ from *in vivo* tissues. Organoids mainly contain epithelial cells and lack vascularization and stromal support, limiting their ability to model complex tissue interaction. Although some models incorporate immune and endothelial cells, they still differ significantly from *in vivo* conditions^192^, ^193^. Besides, studies on interorgan communication during the infection process using organoids remain limited, reflecting the technical challenges in this area^193^. Organoid maturity, especially from inducible pluripotent stem cells (iPSCs), and variability in patient‐derived samples due to biopsy site and patient factors are additional issues^194^. Moreover, as to the structure of the organoids, while ALI culture could improve, it still differs from actual tissue anatomy. Because of these limitations, findings from organoid studies that diverge from clinical outcomes should be interpreted with caution^195^.

It is important to note that results from cell lines, organoids, and *in vivo* models can differ significantly^60^. These variations must be carefully considered when interpreting experimental data and drawing conclusions. Each model system has its strengths and limitations, and discrepancies among them can often provide valuable insights into the complexities of biological systems. Given these considerations, we suggest that organoids should complement, rather than replace, *in vivo* experiments. By using organoids in conjunction with animal models and/or clinical studies, researchers can leverage the strengths of each approach. This multifaceted strategy allows for a more comprehensive understanding of biological processes and can help bridge the gap between laboratory findings and clinical applications.

## ANALYSIS TECHNIQUES USED IN MICROORGANISM–HOST INTERACTION STUDIES

Regardless of the model selected when studying microorganism–host interaction, analytical techniques, particularly sequencing technologies, are essential for elucidating the molecular mechanisms.

**Figure 3:**
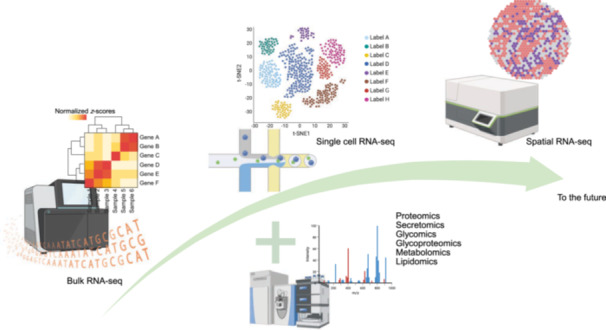
Schematic illustration of analytical techniques for studying host–microbe interactions. Approaches have progressed from bulk RNA sequencing to single cell resolution, alongside the development of multi‐omics integration and spatial transcriptomics. However, limitations remain, highlighting the need for further methodological advances. This figure was created in Bio‐Render. Li, L. (2026) https://BioRender.com/kq7kjkw.

RNA sequencing (Figure 3), capable of simultaneous monitoring of gene expression in both hosts and pathogens, has become a powerful tool for host–microbe interaction due to its sensitivity and cost‐efficiency compared to microarray and PCR techniques traditionally used^196^. This technique is particularly valuable for studying intracellular pathogens and is often used for temporal studies of bacterial gene expression or comparative studies of different bacterial strains^197^. However, because bacterial and host cells are not homogeneous, bulk sequencing can average out the variability among cells, potentially obscuring important details. Single‐cell RNA sequencing (scRNA‐seq) addresses this issue by providing insights into cell‐to‐cell variability. Various scRNA‐seq platforms, such as SMART‐seq, CEL‐Seq, 10× Genomics, Drop‐seq, MARS‐seq, Seq‐Well, and Fluidigm C1, are used to study host–pathogen interactions^198^, ^199^. The goal of “dual” scRNA‐seq is to profile gene expression for both bacteria and host cells within single infected cells. However, challenges remain, such as bacterial lysis, the low abundance of pathogen RNA compared to the abundance of eukaryotic RNA, and bacterial mRNA capture. While techniques like scRandom‐seq have been proposed^200^, they are still in the early stages of development and not yet widely accessible^199^.

When studying infections in 3D or multicellular environments, spatial information is crucial for understanding microbial infection processes. Platforms like APEX2‐seq, TSA‐seq, laser capture microdissection (LCM), FISSEQ, 10× Visium, Slide‐seq, HDST, Light‐seq, GeoMx, and Pick‐Seq offer spatial sequencing for host gene expression but the ability to detect prokaryotic transcripts is limited^201^, ^202^. For example, in a PA‐infected corneal tissue sample analyzed with the Nanostring GeoMx platform, the host transcriptome was well captured, while only about 100 PA genes were detected^203^. Despite this limited bacterial gene coverage, integrating the host and bacterial transcriptional maps provided valuable insights into the host–pathogen interface.^203^ This approach revealed differentially enriched bacterial transcripts and helped identify novel pathoadaptive mechanisms. It was found that iron acquisition pathogen‐specific gene transcripts showed significant enrichment at the host–pathogen interface, and a novel virulence mediator, PA2590, was also involved in the infection process^203^. Other platforms, such as spatial host‐microbiome sequencing (SHM)‐seq and spatial metatranscriptomics (SmT), provide simultaneous host and microbial gene profiling, though with limited spatial resolution^204^, ^205^. These advancements in spatial sequencing technologies have greatly enhanced our ability to understand the complex spatial dynamics of host–pathogen interactions, providing insights into how gene expression varies across different regions of infected tissues or organoids. However, the limitations in prokaryotic transcript detection highlight the ongoing challenges in fully capturing the spatial aspects of microbial gene expression during infection processes.

Transcriptomic data alone may not reflect protein abundance, which is where proteomics can help. Traditional proteomics methods (e.g., 2‐DE, MuDPIT, ICAT, iTRAQ, SILAC, and SELDI‐TOF MS) and newer spatial proteomics tools (e.g., BioID, TurboID, APEX, and APEX2) improve local protein analysis. Microscopy‐based spatial omics, including LCM, deep visual proteomics (DVP), and autoSTOMP, also contribute to this field^201^, ^206^. The integration of proteomics with transcriptomics provides a more comprehensive understanding of host–pathogen interactions, allowing researchers to correlate changes in gene expression with actual protein levels and localization. This multi‐layered approach offers valuable insights into the functional consequences of gene expression changes during infection.

The study of host–pathogen interactions involves not just mRNA and proteins but also genomics, epigenomics, glycomics, and metabolomics. Integrating multi‐omics approaches—encompassing genomics, transcriptomics, epigenomics, proteomics, secretomics, glycomics, glycoproteomics, metabolomics, and lipidomics—can provide a comprehensive view of the interaction process and improve our understanding of its complexities, especially when these approaches include spatial resolution^207^ (Figure 3). This holistic approach to studying host–pathogen interactions allows researchers to capture the full spectrum of molecular changes occurring during infection, from genetic alterations to metabolic shifts. By combining these diverse omics techniques, scientists can build a more complete picture of the intricate interplay between hosts and pathogens, potentially revealing new targets for therapeutic interventions or diagnostic markers. For example, when comparing host responses to *Salmonella* Typhi (human‐specific) and *Salmonella* Typhimurium (broad host range), transcriptomic, proteomic, and metabolomic analyses of mouse spleen were performed. Paired distribution analysis of transcriptomic and proteomic fold changes revealed strain‐specific patterns linked to metabolic pathways. By integrating transcriptomic with metabolomic data, and further combining proteomic with metabolomic data sets, unique metabolic processes associated with *S*. Typhi were discovered, that is, *S*. Typhi infection redirects glycolytic flux and alters pyruvate metabolism, leading to increased ROS production and modulation of immune responses^208^. Although the use of multi‐omics on host–pathogen interactions has mainly focused on bulk sequencing so far, future incorporation of spatial resolution into multi‐omics approaches is expected to improve our ability to localize molecular changes within infected tissues or organoids, thereby providing important context for understanding infection progression and spread.

While individual analytical pipelines and integrative omics analyses offer powerful tools for understanding host–pathogen interactions, they also present significant challenges. Each omic approach has its own technical hurdles, such as sample preparation, data acquisition, and bioinformatics analysis. For instance, in proteomics, issues like protein degradation, post‐translational modifications, and the wide dynamic range of protein abundance can complicate accurate quantification. Transcriptomics faces challenges in capturing low‐abundance transcripts and distinguishing between host and pathogen RNA, especially in cases of low microbial load. Metabolomics struggle with the identification of unknown metabolites and the rapid turnover of certain metabolic intermediates. When it comes to integrative omics, the complexity increases exponentially. Combining data from different omics platforms often involves dealing with varying data formats, different scales of measurement, and the need for sophisticated statistical methods to identify meaningful correlations across diverse data sets. There is also the challenge of temporal and spatial alignment of data from different omics experiments, as well as accounting for the inherent biological variability in host–pathogen systems. Furthermore, the sheer volume of data generated by multi‐omics approaches requires significant computational resources and expertise in data integration and interpretation. Developing standardized protocols for data integration, improving computational tools for cross‐omics analysis, and enhancing our biological understanding to interpret complex, multi‐dimensional data remain ongoing challenges in the field of integrative omics for host–pathogen interaction studies.

In recent years, artificial intelligence (AI) has been increasingly applied in scientific research^209^. In the study of microorganism–host interactions, AI is expected to accelerate research in both data collection and data interpretation. With AI‐assisted engineering, data collection techniques are expected to become more sophisticated, yielding richer information from existing samples. On the interpretation side, experiments now generate vast data sets through various characterization methods such as imaging, multi‐omics (RNA, protein, and metabolite analyses), and sensor‐based measurements. Processing these growing volumes of data manually are both labor‐intensive and time‐consuming. By applying well‐designed models and training strategies, AI can help interpret complex data sets and uncover insights that might be inaccessible through traditional approaches. Since microbiota themselves form complicated communities, their interactions with the human host, another complex system, are even more challenging to analyze. Although there have been attempts to understand and predict human–microbiome interactions, our understanding still remains limited^210^, ^211^. Given the scale and complexity of the data involved, AI‐assisted analysis is expected to play an increasingly critical role in this field.

## CONCLUDING REMARKS

In this review, we provide a comprehensive overview of the various models used to study microorganism–host interactions, particularly organoid models. Each model has its own set of advantages and limitations, making the careful selection of the appropriate model crucial for effective research. We have systematically detailed how organoids enhance the study of host–pathogen interactions and highlighted their current limitations. Additionally, we briefly review the analytical techniques used to uncover the underlying molecular mechanisms in these models. This comprehensive approach allows researchers to make informed decisions when designing experiments to investigate host–pathogen interactions, ensuring that the chosen model and analytical techniques align with the specific research questions being addressed.

While various pathogens have been clinically identified, research on many of them remains limited, likely due in part to the challenge of finding suitable models. Currently, organoid models, such as brain, heart, and liver organoids, have primarily been utilized in viral infection studies and have seen limited application in bacterial research. However, as organoid technologies continue to advance, becoming more complex and physiologically accurate, they hold the potential to significantly expand our ability to study a broader range of infections.

The study of host–pathogen interactions faces challenges not only from the models used but also from the analytical techniques available. We anticipate that ongoing advancements in both organoid development and analytical methodologies will greatly enhance our understanding of microorganism–host dynamics, paving the way for new insights and therapeutic approaches.
